# Lycobetaine Has Therapeutic Efficacy in Lung Squamous Cell Carcinoma by Targeting USP32 to Trigger Ferroptosis

**DOI:** 10.3390/cimb47030163

**Published:** 2025-02-27

**Authors:** Shangping Xing, Hua Chai, Zhenlong Chen, Shuye Deng, Feifei Nong

**Affiliations:** 1 Pharmaceutical College, Guangxi Medical University, Nanning 530021, China; shopingxing@sr.gxmu.edu.cn (S.X.); 20224061727@sr.gxmu.edu.cn (H.C.); 20224061701@sr.gxmu.edu.cn (Z.C.); 2 Guangxi Key Laboratory for Bioactive Molecules Research and Evaluation, Nanning 530021, China; 3 Guangxi Key Laboratory of Pharmaceutical Precision Detection and Screening, Nanning 530021, China; 4 Department of Scientific Research, The First Affiliated Hospital of Guangxi University of Chinese Medicine, Guangxi University of Chinese Medicine, Nanning 530024, China

**Keywords:** lycobetaine, ferroptosis, USP32, NRF2, LUSC, ubiquitination

## Abstract

Ubiquitin-specific protease 32 (USP32), a deubiquitylating enzyme that controls the ubiquitin process, is overexpressed in multiple cancers and serves as a promising therapeutic target for cancer therapy. Drugs targeting ferroptosis have exhibited promising anticancer activity. Lycobetaine (LBT), a natural alkaloid, holds promise against various cancers, yet its specific targets and anticancer mechanisms remain unclear. In this study, we show that LBT induced ferroptosis in lung squamous cell carcinoma (LUSC) cells, accompanied by glutathione depletion and the accumulation of lipid peroxidation, malondialdehyde, and ferrous iron. Mechanistically, drug affinity responsive target stability-based mass spectrometry analysis, molecular dynamics simulations, and a cellular thermal shift assay confirmed that USP32 is a potential target of LBT in LUSC cells. Moreover, a strong interaction between USP32 and nuclear factor erythroid 2-related factor 2 (NRF2) was found via immunoprecipitation–mass spectrometry and co-immunoprecipitation. In addition, the ubiquitination assay results demonstrated that LBT treatment significantly increased NRF2 ubiquitination and degradation by targeting USP32. Importantly, USP32 overexpression effectively attenuated the effects of LBT on proliferation and ferroptosis in LUSC cells. In orthotopic LUSC xenografts, the administration of LBT significantly inhibited tumor growth and metastasis and induced ferroptosis by targeting the USP32–NRF2 signaling axis. Taken together, these data suggest that LBT exerts its anticancer effects by inhibiting USP32-mediated NRF2 deubiquitination to induce ferroptosis and that LBT may serve as a prospective USP32-targeting agent for LUSC treatment.

## 1. Introduction

Lung cancer is the most common cancer and remains the leading cause of cancer-associated mortality worldwide [1]. Approximately 85% of lung cancer is non-small-cell lung cancer (NSCLC), which is mainly classified into lung adenocarcinoma (LUAD) and lung squamous cell carcinoma (LUSC). Unlike LUAD, for which targeted therapies, including epidermal growth factor receptor and anaplastic lymphoma kinase inhibitors, have shown significant efficacy, patients with LUSC have not benefited from targeted therapies [2]. Therefore, the identification of effective therapeutics for patients with LUSC is urgently needed.

Proteolysis by the ubiquitin–proteasome system (UPS) is required for the maintenance of cellular protein homeostasis via cooperation between ubiquitination and deubiquitination among eukaryotes [3]. Ubiquitination is a reversible post-translational modification involving the covalent linking of ubiquitin (Ub) to targeted substrates by the Ub ligase system (E1–E2–E3) in eukaryotes [4]. Ubiquitination is vital for protein degradation and is implicated in serious diseases, including cancers [5]. However, ubiquitination can be reversed by deubiquitinating enzymes (DUBs), which further reverses UPS-mediated degradation [6]. Therefore, it is now widely accepted that DUBs play an important regulatory role in tumorigenesis and cancer development [7,8]. The ubiquitin-specific peptidase 32 (USP32), a member of the USP family of DUBs, is a membrane-bound deubiquitinating enzyme, which was first discovered to act as an oncogene in breast cancer [9]. Accumulating evidence has demonstrated that USP32 is overexpressed in various tumors and is significantly involved in tumor development, cancer-related signaling, and protein stability [10,11]. USP32 participates in tumor growth and resistance to therapy via the deubiquitination modification of tagged substrates, such as Rap1b and SLC35F2, suggesting that USP32 is a potential therapeutic target [12,13]. Ferroptosis is an iron-dependent form of regulated cell death, arising from uncontrolled and excessive lipid peroxidation [14]. In recent years, ferroptosis has garnered enormous interest in cancer research communities as targeting ferroptosis may hold great potential for cancer therapy [15]. The nuclear factor erythroid 2-related factor 2 (NRF2) is the master regulator of cellular antioxidant properties and ferroptosis protection [16]. DUBs may play an important role in ferroptosis by altering substrate stability [17]. One previous study also suggested that the pharmacological inhibition of USP8 can induce ferroptosis and inhibit the progression of hepatocellular carcinoma by decreasing the stability of the O-GlcNAcylation of solute carrier family 7 member 11 (SLC7A11) [18]. USP13 has been reported to facilitate ferroptosis by activating NRF2 in LUAD [19]. Therefore, it has not been ruled out that USP32 may be involved in regulating ferroptosis in LUSC by controlling the degradation of proteins related to iron homeostasis, including NRF2, SLC7A11, and glutathione peroxidase 4 (GPX4).

Lycobetaine (LBT), also known as ungeremine, is a quaternary phenanthridinium alkaloid that can be extracted from several medicinal herbs from the Amaryllidaceae family [20]. LBT acts as a specific topoisomerase IIβ poison and has been studied extensively in a range of cancer cell types in vitro and tumor types in vivo, such as LXFS538, CXF280, PAXF546, MCF7X, and LXFA526 cells, as well as 21 human tumor xenografts [21]. It has also been reported that LBT induces reactive oxygen species (ROS) production, apoptosis, necroptosis, ferroptosis, and autophagy in CCRF-CEM leukemia cells [22]. However, the effects of LBT on LUSC and its molecular mechanisms still remain unknown, which guided the aim of this study. In this study, we investigated the effects of LBT on the cell viability of two different LUSC cell lines and found that LBT induced ferroptosis in LUSC cells. Next, the anticancer targets of LBT were explored using a drug affinity responsive target stability (DARTS)-based mass spectrometry analysis (MS) strategy, and USP32 was identified as the direct target of LBT, which has not yet been reported. Subsequently, the immunoprecipitation–MS (IP-MS) technique was used to identify the binding ferroptosis-related proteins of USP32, and we showed that the USP32–NRF2 axis is a critical mediator in LBT-induced ferroptosis. Taken together, these data could unveil novel targets and mechanisms underlying LBT-induced ferroptosis in LUSC.

## 2. Materials and Methods

### 2.1. Reagents and Antibodies

LBT was purchased from Yuanye Biological Co., Ltd. (Shanghai, China). Z-VAD, ferrostain-1 (Fer-1), necrostain-1 (Nec-1), and chloroquine (CQ) were purchased from MedChemExpress (Monmouth Junction, NJ, USA). The recombinant lentivirus targeting USP32 for humans was purchased from GenePharma (Suzhou, China). The antibodies against GPX4, SLC7A11, Transferrin, USP32, NRF2, NQO1, and GAPDH were purchased from Cell Signaling Technology (Beverly, MA, USA). The antibodies against Myc-Tag, Flag (DDDDK)-Tag, and HA-Tag were purchased from ABclonal (Wuhan, China).

### 2.2. Cell Culture and Transfection

The LUSC cell lines H520 and H1703 and the human kidney cell line 293T were purchased from the American Type Culture Collection (Manassas, VA, USA). The cell lines were cultured in RPMI-1640 medium, supplemented with 10% FBS, penicillin, and streptomycin (all from Gibco, Grand Island, NY, USA), at 37 °C in a 5% CO_2_ incubator. The stable cell lines were established as previously described [23]. The H520 and H1703 cells were infected with a lentivirus targeting USP32 for 48 h. Then, the cells were incubated with a medium containing puromycin (3 µg/mL) to generate cells that stably overexpressed USP32.

### 2.3. Cell Viability Assay

The LUSC cells (5 × 10^3^/well) were seeded in 96-well plates and treated with LBT, Z-VAD, Fer-1, Nec-1, and CQ at the indicated concentrations for 24 h or 48 h. Cell viability was determined by a CCK-8 assay (Dojindo, Kumamoto, Japan), and absorbance at a wavelength of 450 nm was measured using a microplate reader (PerkinElmer, Waltham, MA, USA).

### 2.4. Measurement of Glutathione (GSH), Malondialdehyde (MDA), and Ferrous Iron

According to the manufacturer’s instructions, the levels of GSH, MDA, and ferrous iron in the LUSC cells were measured using MDA and GSH assay kits (Nanjing Jiancheng Bioengineering Institute, Nanjing, China) and an iron colorimetric assay kit (Elabscience, Wuhan, China).

### 2.5. Measurement of Lipid Peroxidation

The total cellular lipid peroxidation was measured using a lipid peroxidation assay kit with BODIPY 581/591 C11 (Beyotime, Shanghai, China). The LUSC cells were incubated with a 2 μM BODIPY 581/591 C11 probe for 30 min at 37 °C in the dark. Then, the lipid peroxidation levels of the LUSC cells were analyzed using flow cytometry (BD, San Jose, CA, USA).

### 2.6. Molecular Docking and Molecular Dynamic Simulations

For molecular docking, the crystal structure of USP32 (AF-Q8NFA0-F1-v4) was obtained from the AlphaFold Protein Structure Database (https://alphafold.com/). The 3D conformer structure of LBT (CID: 159646) was obtained from PubChem (https://pubchem.ncbi.nlm.nih.gov/) in SDF format. The receptor (USP32) and ligand molecules (LBT) were then subjected to molecular docking using AutoDockTools-1.5.6. The best fit complex (LBT–USP32) resulting from this molecular docking was visually analyzed using PyMOL 2.6 software. The molecular dynamic simulations were performed using the GROMACS2020 package and the AMBER99SB force field. The LBT–USP32 protein–ligand complexes, initially derived from the molecular docking, were immersed in a cubic water box, modeled using SPC solvent. The ligand topology parameters were generated by Sobtop 1.0 software. Equilibration involved sequential NVT and NPT ensembles at 300 K and 1.0 atm, respectively, spanning 50 ns with 25 million integration steps. Structural stability was evaluated by calculating the root mean square deviation (RMSD) using the QtGrace 0.2.6 software.

### 2.7. DARTS-MS Analysis

H520 cells were lysed in an NP-40 lysis buffer (Beyotime) mixed with a 1% protease inhibitor cocktail (MedChemExpress). The cell lysates were centrifuged at 12,000 rpm for 20 min at 4 °C and then the supernatant was collected and incubated with LBT or a solvent control (DMSO) for 1 h at room temperature. After incubation with pronase (MedChemExpress) at room temperature for 30 min, digestion was terminated using a protease inhibitor for 10 min. The samples were separated by SDS-PAGE and then the gel bands were cut out and digested with trypsin. The resulting peptides were analyzed by Nano LC-MS/MS (Thermo Fisher Scientific, Waltham, MA, USA). The proteins were identified from the MS/MS spectra using Proteome Discoverer software (version 2.2; Thermo Fisher Scientific).

### 2.8. IP-MS Analysis

H520 cells were transfected with Flag-tagged USP32 using a Lipofectamine 3000 kit (Thermo Fisher Scientific) and then lysed in the NP-40 lysis buffer (Beyotime) mixed with the 1% protease inhibitor cocktail (MedChemExpress). The cell lysates were centrifuged, and the supernatant was incubated with anti-FLAG M2 agarose (Sigma-Aldrich, St. Louis, MO, USA) for 2 h at 4 °C to enrich USP32-binding proteins. The proteins in the agarose were digested with trypsin, followed by Nano LC-MS/MS (Thermo Fisher Scientific). The proteins were identified from the MS/MS spectra using Proteome Discoverer software (version 2.2; Thermo Fisher Scientific).

### 2.9. Western Blotting and Co-IP

The cells were lysed in RIPA buffer mixed with a 1% protease inhibitor cocktail (MedChemExpress), and the protein samples were separated by SDS-PAGE and transferred onto PVDF membranes (Millipore, Burlington, MA, USA). After blocking in 5% non-fat milk for 1 h at room temperature, the membranes were further incubated with the primary antibodies overnight at 4 °C and incubated with corresponding secondary antibodies for an additional 1 h at room temperature. The immunoreactive protein bands with an ECL reagent (Millipore) were acquired and analyzed using the Tanon 5200 image acquisition system (Shanghai, China). For IP, the protein lysates were extracted using an IP lysis buffer (Beyotime). The protein supernatants were collected via centrifugation and incubated with antibodies against Immunoglobulin G (IgG) and USP32 overnight at 4 °C, followed by incubation with the protein A + G agarose (Beyotime) for 4 h at 4 °C. The protein A + G agarose was washed five times in the IP buffer and then boiled with an SDS-PAGE loading buffer for Western blot analysis.

### 2.10. In Vivo Ubiquitination Assay

This assay was performed as previously described [24]. Briefly, the 293T cells were co-transfected with Flag-USP32, Keap1, HA-Ub, and MyC-NRF2 using a Lipofectamine 3000 kit (Thermo Fisher Scientific). After 48 h, the cells were treated with or without LBT for 24 h and then treated with MG132 for an additional 6 h. The cells were then evaluated via immunoprecipitation and Western blot analysis.

### 2.11. Cellular Thermal Shift Assay (CETSA)

H520 cells were treated with LBT or a solvent control (DMSO) for 3 h. The cells were then digested with trypsin and resuspended in PBS. The cell suspensions were divided into six PCR tubes and heated at a range of temperatures (25, 42, 46, 50, 54, and 58 °C) for 3 min. Then, the tubes underwent three freeze–thaw cycles in liquid nitrogen. Subsequently, the supernatant was collected via centrifugation at 12,000 rpm for 20 min at 4 °C and studied via Western blot analysis.

### 2.12. Orthotopic Lung Cancer Models

All animal studies were approved by the Institutional Animal Care and Use Committee of Guangxi Medical University (Approval No. 202402002). The animal studies were conducted in compliance with the ARRIVE guidelines. BALB/c nude mice (5-week-old males) were purchased from Guangxi Medical University and raised in a specific pathogen-free laboratory. To establish the orthotopic LUSC model, H520 cells suspended in Matrigel (BD; 1:1 ratio in volume) were orthotopically injected into the pleura of the mice. After 10 days, orthotopic LUSC tumors were confirmed following the dissection of mice from the same batch. The orthotopic LUSC tumor-bearing mice were randomly divided into three groups to treat with vehicles, LBT (10 mg/kg), and LBT (20 mg/kg). After 18 days of treatment (intraperitoneal injections once a day), the mice were sacrificed and the orthotopic and metastatic lung nodules were evaluated via H&E staining and ferroptosis analysis.

### 2.13. H&E Staining

The lung cancer tissues were fixed in 4% paraformaldehyde. The samples were then dehydrated, embedded with paraffin, and sectioned to a 4 μM thickness. Finally, the paraffin sections were processed with an H&E staining kit (Beyotime), according to the manufacturer’s instructions.

### 2.14. Statistical Analysis

Experimental values are presented as the mean ± SD using GraphPad Prism 9.0 (GraphPad Software, San Diego, CA, USA). Statistical analyses were performed using SPSS 22.0 (SPSS Inc., Chicago, IL, USA). One-way analysis of variance (ANOVA) or unpaired *t*-test was applied to determine statistical significance. Values of *p* < 0.05 indicated statistical significance.

## 3. Results

### 3.1. Ferroptosis Is Associated with LBT-Induced Suppression in LUSC Cells

**Figure 1:**
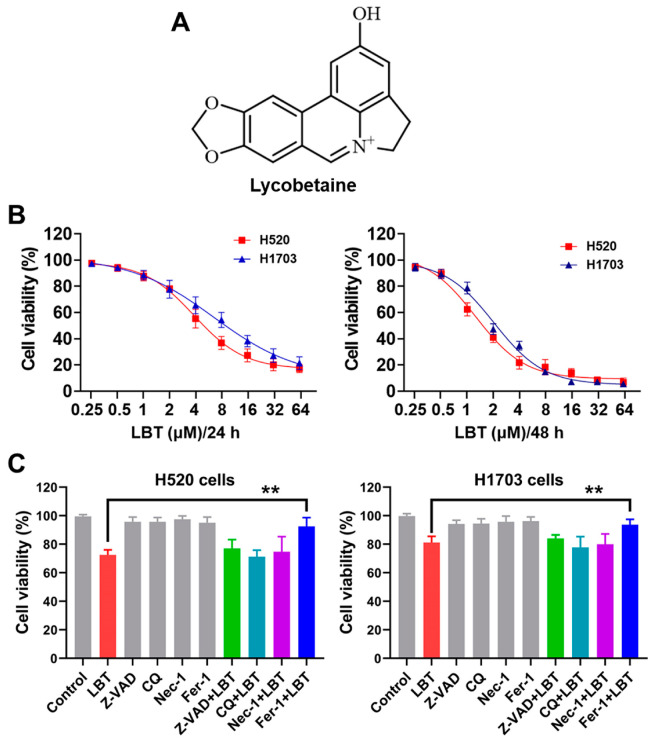
(**A**) The chemical structure of LBT; (**B**) the cytotoxicity of LBT toward H520 and H1703 cells, determined by a CCK-8 assay; (**C**) the cell viability of H520 and H1703 cells treated with LBT (1 µM), Z-VAD (10 µM), CQ (25 µM), Nec-1 (20 µM), and Fer-1 (1 µM), determined by a CCK-8 assay. The results are presented as mean ± SD. ** *p* < 0.01 versus the control group.

The cytotoxic effects of LBT (Figure 1A) on LUSC cells were first evaluated using a CCK-8 assay. As shown in Figure 1B, LBT significantly inhibited the growth of H520 and H1703 cells in a dose- and time-dependent manner. The IC_50_ values of LBT on the H520 and H1703 cells at 48 h were 1.9 and 2.2 µM, respectively. To determine what kind of cell death was induced by LBT, several cell death inhibitors were utilized. As shown in Figure 1C, LBT-induced cell death was attenuated by a ferroptosis inhibitor (Fer-1), while treatment with an apoptosis inhibitor (Z-VAD), autophagy inhibitor (CQ), and necrosis inhibitor (Nec-1) did not protect against LBT-induced cell death in LUSC cells. These results indicated that ferroptosis contributed to LBT-induced suppression in LUSC cells.

### 3.2. LBT Induces Ferroptosis in LUSC Cells

**Figure 2:**
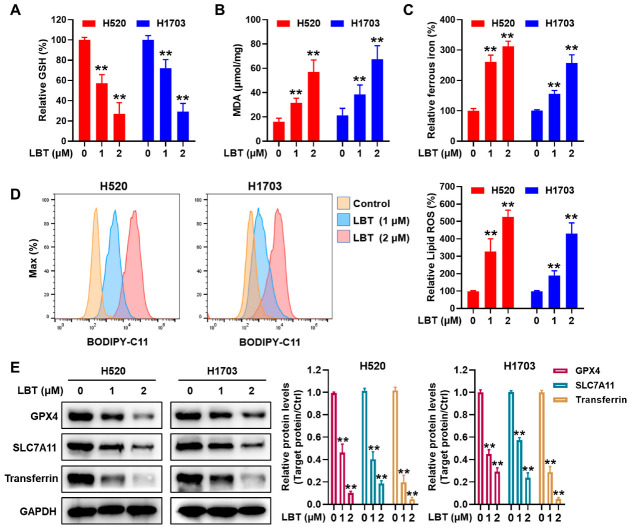
(**A**) The GSH, (**B**), MDA, and (**C**) ferrous iron assays performed after LBT treatment in H520 and H1703 cells; (**D**) the lipid ROS levels, evaluated using a BODIPY 581/591 C11 probe and flow cytometry; (**E**) the protein levels of GPX4, SLC7A11, and transferrin in H520 and H1703 cells, examined by Western blot analysis. The results are presented as mean ± SD. ** *p* < 0.01 versus the control group.

To further verify that LBT induces ferroptosis in LUSC cells, we examined the characteristics of ferroptosis. As shown in Figure 2A–C, the levels of GSH were markedly decreased due to being inhibited by LBT, whereas the levels of MDA and ferrous iron were obviously increased in a dose-dependent manner. Using a BODIPY 581/591 C11 probe and flow cytometry, we confirmed that LBT treatment significantly induced lipid peroxidation (Lipid ROS) accumulation in LUSC cells (Figure 2D). Additionally, Western blot analysis revealed that LBT significantly decreased the expression of ferroptosis-related markers, including GPX4 and SLC7A11, while markedly increasing the expression of transferrin, an iron-related protein, in LUSC cells (Figure 2E). These results demonstrated that ferroptosis induction is the primary effect of LBT against LUSC cells.

### 3.3. Identification of USP32 as a Potential Target of LBT

**Figure 3:**
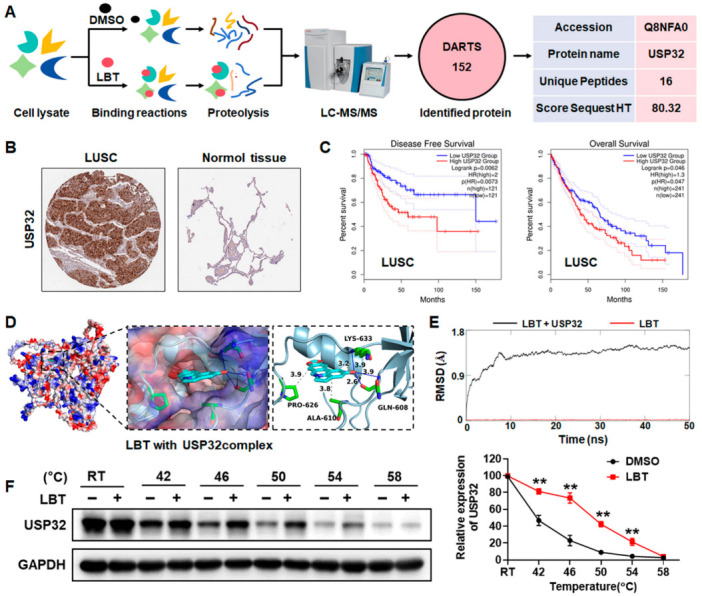
(**A**) A schematic representation of the procedures used to identify LBT-binding proteins in the DARTS-MS analysis; (**B**) the expression of USP32 in LUSC and normal human tissues in IHC samples from the HPA database; (**C**) the correlations between USP32 expression, DFS, and OS in patients with LUSC, analyzed using the GEPIA database; (**D**) a molecular docking model illustrating the binding of LBT to USP32; (**E**) the molecular dynamic simulations for LBT–USP32 complexes; (**F**) the CETSA confirming that LBT targets the USP32 protein. The results are presented as mean ± SD. ** *p* < 0.01 versus the DMSO group.

DARTS-MS analysis was utilized to identify the effects of LBT on LUSC cells. As shown in Figure 3A, 152 proteins were identified as potential targets of LBT, and USP32 was the top enriched protein due to its unique peptide number and HT score. Thus, USP32 was considered a candidate target for further investigation. Moreover, USP32 is highly expressed in human LUSC samples, according to the IHC in the human protein atlas (HPA) database, and is not highly expressed in normal human tissues (Figure 3B). An evaluation of the gene expression profiling interactive analysis (GEPIA) database also found that the disease-free survival (DFS) and overall survival (OS) of LUSC patients with a high expression of USP32 were significantly lower than those with a low expression (Figure 3C), demonstrating that USP32 represents a potential therapeutic target in LUSC.

Interestingly, LBT treatment had no effect on the mRNA expression of USP32 in LUSC cells (Figure S1), indicating that LBT may target USP32. Firstly, molecular docking and molecular dynamic simulations were conducted to estimate the interaction between LBT and USP32. As shown in Figure 3D, LBT was observed to establish three hydrogen bonds with the LYS-633 and GLN-608 residues of USP32, with a binding energy of −8.62 kcal/mol. Furthermore, the RMSD trajectory curve of the small molecule–protein complex (LBT–USP32) remained within a reasonable range (<2.0 Å) and stabilized, indicating a robust interaction between LBT and the USP32 protein (Figure 3E). CETSA was further performed to validate the interaction between LBT and USP32. As expected, the heat challenge quickly led to USP32 denaturation and precipitation, while LBT markedly increased the thermal stability of the USP32 protein at temperatures ranging from 42 °C to 54 °C (Figure 3F), confirming the direct binding of LBT to USP32.

### 3.4. LBT Inhibits USP32-Induced Deubiquitination of NRF2

**Figure 4:**
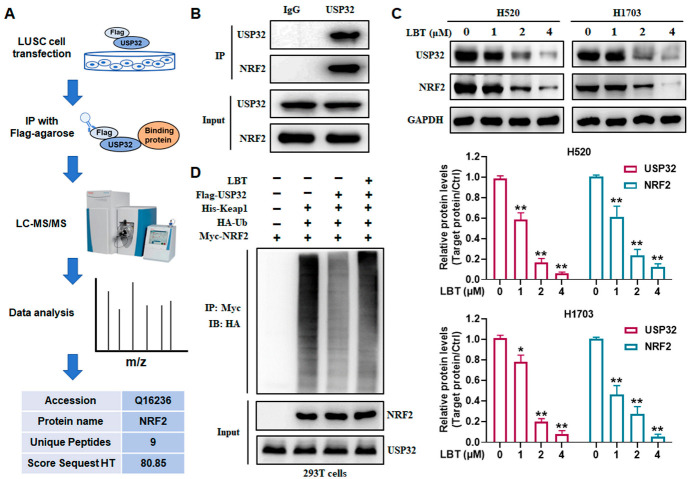
LBT promotes the ubiquitination and degradation of NRF2 through targeting USP32. (**A**) A workflow to identify USP32–NRF2 interaction using IP-MS analysis; (**B**) USP32–NRF2 interaction in H520 cells, examined by Co-IP analysis; (**C**) the protein levels of USP32 and NRF2 in H520 and H1703 cells, examined by Western blot analysis; (**D**) an in vivo NRF2 ubiquitination assay of 293T cells transfected with Flag-USP32, Keap1, HA-Ub, and MyC-NRF2 upon LBT treatment. The results are presented as mean ± SD. * *p* < 0.05 and ** *p* < 0.01 versus the control group.

To investigate the underlying mechanisms of USP32 that regulate ferroptosis in LUSC, an IP-MS assay was applied to further explore the binding proteins of USP32. After the H520 cells were transfected with Flag-USP32, the interacting proteins of USP32 were pulled down with FLAG-agarose for MS analysis. Among the identified binding proteins, NRF2 (a ferroptosis inhibitory protein) was found to bind specifically to USP32 (Figure 4A). Consistently, a GEPIA database analysis found that the expression of USP32 and NRF2 exhibited a strong positive correlation in patients with LUSC (Figure S2). Furthermore, the results of the Co-IP analysis also revealed a strong interaction between USP32 and NRF2 (Figure 4B). Additionally, we found that LBT treatment obviously decreased the protein expression levels of USP32 and NRF2 in LUSC cells (Figure 4C). The Western blot results also demonstrated that LBT significantly suppressed NQO1 expression, a key downstream target of NRF2, in LUSC cells, consistent with NRF2 inhibition (Figure S3). The ubiquitination degradation of NRF2 depends on the ubiquitination mark of Keap1-Cul3 E3 ligase [25]. Thus, we further assessed whether USP32 could inhibit the ubiquitination of NRF2 caused by Keap1 and whether LBT could promote the ubiquitination of NRF2 by targeting USP32. The results demonstrated that USP32 markedly inhibited Keap1-mediated NRF2 ubiquitination, whereas LBT treatment effectively reversed the deubiquitination of NRF2 mediated by USP32 (Figure 4D). These results demonstrated that LBT promotes the ubiquitination and degradation of NRF2 by targeting USP32, thereby inducing ferroptosis in LUSC cells.

### 3.5. USP32 Overexpression Reverses the LBT-Induced Ferroptosis of LUSC Cells

**Figure 5:**
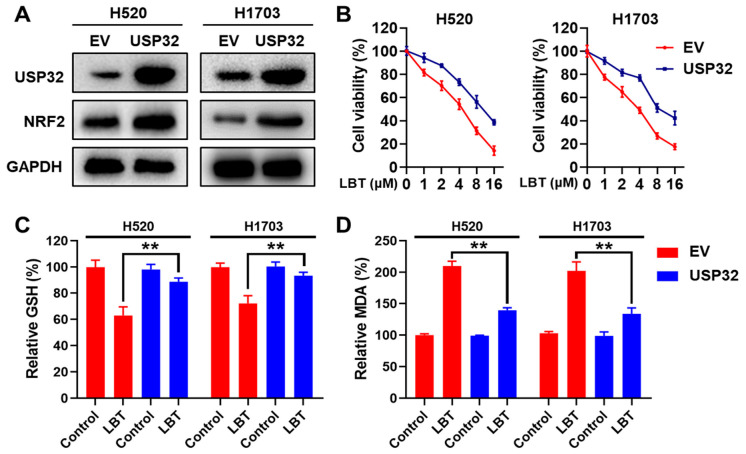
LBT promotes ferroptosis by targeting USP32 in LUSC cells. (**A**) The protein levels of USP32 and NRF2 in H520 and H1703 cells that stably overexpressed USP32 or empty vector (EV), examined by Western blot analysis; H520 and H1703 cells with USP32 overexpression or EV that were treated with LBT, analyzed using (**B**) a CCK-8 assay, (**C**) a GSH assay, and (**D**) an MDA assay. The results are presented as mean ± SD. ** *p* < 0.01 versus the EV group.

To validate whether LBT induces ferroptosis by targeting USP32, we established the stable overexpression of USP32 in H520 and H1703 cells using lentivirus infection. We found that USP32 overexpression increased NRF2 protein levels in LUSC cells (Figure 5A). A CCK-8 assay revealed that USP32 overexpression obviously reversed the inhibitory effects of LBT on cell viability in LUSC cells (Figure 5B). Consistently, LBT-induced reductions in GSH levels and increases in MDA levels were significantly attenuated by USP32 overexpression in LUSC cells (Figure 5C,D). These results strongly confirmed that USP32-targeting contributes to LBT-induced ferroptosis in LUSC.

### 3.6. LBT Induces Ferroptosis in LUSC In Vivo

**Figure 6:**
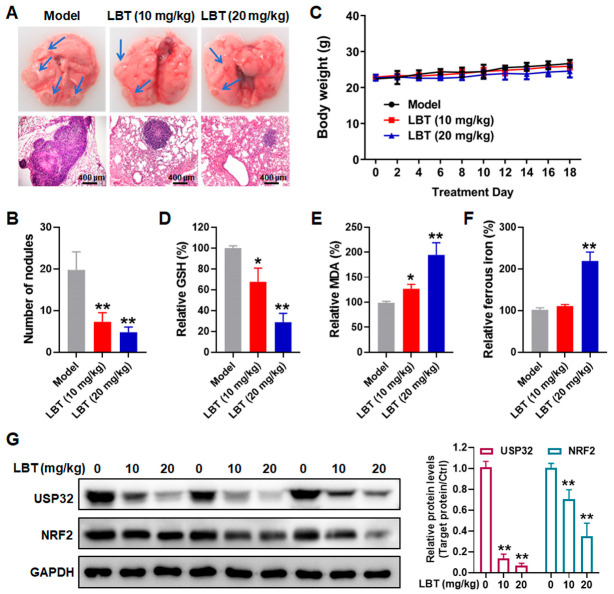
LBT triggers ferroptosis in LUSC orthotopic model. (**A**) Images of lungs harvested from mice after 18 days of LBT treatment (upper panel), and hematoxylin–eosin (H&E) staining of metastatic nodules in lung tissues (lower panel) (blue arrows indicate tumor tissue areas; scale bar: 400 µm); (**B**) number of metastasis nodules in the lung tissues; (**C**) body weight and levels of (**D**) GSH, (**E**) MDA, and (**F**) ferrous iron in each group; (**G**) protein levels of USP32 and NRF2 in tumors, examined by Western blot analysis. The results are presented as mean ± SD. * *p* < 0.05 and ** *p* < 0.01 versus the model group.

We established an orthotopic model of LUSC to assess the therapeutic potential of LBT in inducing ferroptosis in vivo. Our results demonstrated that LBT treatment significantly reduced the size and number of metastatic lung nodules compared to the model group (Figure 6A,B). Of note, LBT treatment did not cause any body weight loss (Figure 6C) or hepatorenal toxicity, as evidenced by the lack of statistically significant differences in ALT, AST, and CRE levels between the model and LBT-treated groups (Figure S4). Further analysis revealed that LBT decreased GSH levels and increased MDA and ferrous iron production compared to the model group (Figure 6D–F). Consistent with the in vitro results, LBT administration significantly decreased the protein expression levels of USP32 and NRF2 in tumor tissues (Figure 6G). Taken together, these results revealed that LBT triggered ferroptosis in LUSC cells in vivo by targeting the USP32–NRF2 axis.

## 4. Discussions

LUSC accounts for more than 30% of NSCLC, with a dismal prognosis that lacks adequate therapies and actionable targets [26]. To date, immunotherapy has evolved into a successful therapeutic strategy for patients with LUSC, but the overall response rate remains low [27]. Therefore, identifying potent drugs and biomarkers for targeted therapies represents an urgent unmet need for patients with LUSC.

Over the past five years, the approval rate of natural product-derived drugs by the US Food and Drug Administration has consistently exceeded 20% [28]. Accumulating evidence has indicated that natural products exhibit promising antitumor activities [29,30]. LBT, a natural metabolite of lycorine, has demonstrated anticancer activity, low toxicity, and no immunosuppression [31,32]. However, the role of LBT in LUSC treatment remains unclear. In this research, we observed the effects of LBT on LUSC cells both in vitro and in vivo. The results showed that LBT could inhibit the cell viability of H520 and H1703 cells and suppress tumor growth and metastasis in an orthotopic LUSC model, without any apparent toxic side effects. Intriguingly, LBT-induced cell death in LUSC cells was blocked by the ferroptosis inhibitor Fer-1, suggesting that LBT-induced cell death may occur via ferroptosis. Accumulating evidence has suggested that cancer cells that are resistant to chemotherapy, targeted therapies, and immunotherapy may be susceptible to ferroptosis; thus, targeting ferroptosis may provide new therapeutic opportunities for treating LUSC [33,34,35]. Ferroptosis is mainly caused by iron-dependent lipid peroxidation; thus, potent initiators of lipid peroxidation, such as ROS and lipid peroxidation products (e.g., MDA), are powerful inducers of ferroptosis [36]. Our data showed that ferroptosis events, including GSH depletion and ferrous iron, MDA, and lipid peroxidation (Lipid ROS) accumulation, were significantly triggered following treatment with LBT. Multiple studies have provided evidence that the SLC7A11–GSH–GPX4 axis is the major cellular system defending against ferroptosis. The genetic or pharmacological inhibition of GPX4 and SLC7A11 causes GSH depletion and lipid peroxidation, resulting in ferroptotic cell death [37]. After treatment with LBT, we found that the protein expression of GPX4 and SLC7A11 was significantly decreased in LUSC cells. These findings confirmed that ferroptosis contributes to LBT-induced growth inhibition in LUSC cells.

MS-based proteomic approaches are among the most effective tools for identifying the targets of natural products [38]. In our previous study, skp1 was identified as the antitumor target of brusatol via a streptavidin-affinity pull down-MS analysis [24]. The DARTS technique, which is based on drug binding-incurred changes in the protease susceptibility of a target protein, requires no modifications to drugs and is independent from the mechanisms of drug actions [39]. Thus, to determine how LBT induces ferroptosis in LUSC cells, the DARTS-MS method was first utilized for target prediction and found that LBT may target USP32, a universal oncogene in a variety of tumors. USP32 has been reported to enhance the transmission of imatinib resistance by inhibiting the ubiquitin–proteasome system [40]. USP32 promotes the malignant behaviors of acute myeloid leukemia cells by regulating the stability of Rap1b [12]. In addition, high USP32 expression plays a crucial role in tumor growth, metastasis, immune infiltrates, and chemoresistance via the deubiquitination of various substrate proteins, such as Smad2, SHMT2, FDFT1, Rab7, SLC35F2, and BAG3 [10,41,42,43,44,45]. Thus, USP32-specific inhibitors are promising therapeutic drugs for tumor therapies. In this study, we performed an in-cell CETSA experiment and found that LBT possessed a direct interaction with USP32 by affecting thermal stability, which was consistent with the DARTS results. In addition, given the evidence that USP32 overexpression significantly reversed LBT-induced ferroptosis in LUSC cells, our study provided evidence that USP32 is an essential target of LBT.

However, the influence of USP32 on ferroptosis in cancer cells remains unclear. Here, an IP-MS analysis was also conducted to identify USP32-binding ferroptosis-related proteins, and the transcription factor NRF2 drew our attention. Moreover, the Co-IP results indicated the direct association of USP32 with NRF2, suggesting that USP32 may be a deubiquitinase of NRF2. Under normal physiological conditions, NRF2 undergoes Keap1-cullin 3 E3 complex-dependent ubiquitination and proteasome degradation in cytoplasm, thereby maintaining its low-level expression. Nevertheless, in tumor cells, Keap1 inactivation leads to an increase in the stability and activity of NRF2, subsequently activating numerous target genes involved in GPX4–GSH-mediated ferroptosis defense, including SLC7A11, thereby facilitating the protection of cancer cells from ferroptosis [46]. NRF2 is overexpressed in a number of cancer types, such as LUSC, colorectal cancer, and hepatocellular carcinoma, and is associated with poor prognosis for cancer patients [16]. As a master regulator of antioxidant defense, numerous studies have demonstrated that NRF2 plays a pivotal role in regulating ferroptosis via a number of proteins whose functions are closely tied to the ferroptosis cascade, such as GSH synthesis/metabolism and lipid metabolism, which are NRF2 target genes [47]. Many of the currently identified natural products induce ferroptosis in tumors via the NRF2 pathway [48,49]. Our recent study also found that penexanthone A, a xanthone dimer component derived from marine fungi, induces ferroptosis by inhibiting the NRF2 pathway in colorectal cancer cells [23]. Consequently, the pharmacological modulation of NRF2 to induce ferroptosis is a promising area of interest.

**Figure 7:**
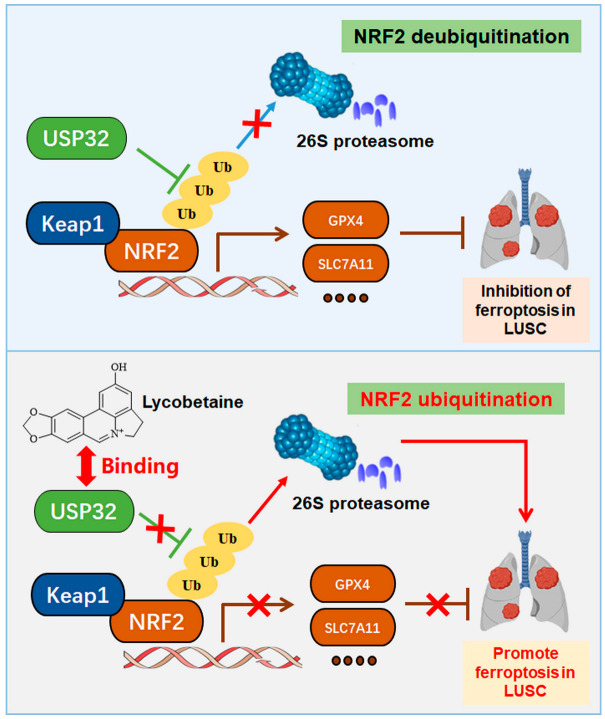
A schematic representation of the mechanisms by which LBT induces ferroptosis in LUSC cells. Top panel: USP32-mediated NRF2 deubiquitination suppressing ferroptosis. Bottom panel: LBT targets USP32 to promote NRF2 ubiquitination and degradation, inducing ferroptosis. “↓” indicates promotion; “⊥” indicates inhibition.

As a pivotal regulatory system for protein homeostasis, DUBs substantially impact ferroptosis by controlling the ubiquitination and stability of ferroptosis-related proteins [17,50]. Studies have shown that the DUB USP11 can bind and promote the deubiquitination modification of NRF2, thereby conferring resistance to ferroptosis in lung cancer cells [51]. In addition, DHPO has been identified as a potent USP7 inhibitor, which has been found to induce ferroptosis in gastric cancer cells by targeting the deubiquitination of Stearoyl-CoA Desaturase mediated by USP7 [52]. Therefore, we tried to demonstrate whether USP32 inhibits the ubiquitination of NRF2 caused by Keap1 and whether LBT enhances the ubiquitination and degradation of NRF2 by targeting USP32 to induce ferroptosis. In our study, we found that USP32 markedly inhibited Keap1-mediated NRF2 ubiquitination and that USP32 overexpression markedly increased NRF2 protein levels, which demonstrated that USP32 is a DUB of NRF2. Meanwhile, LBT effectively inhibited USP32-mediated NRF2 deubiquitination and the protein levels of USP32 and NRF2, whereas USP32 overexpression markedly reversed the inhibitory effects on LUSC cell proliferation and the occurrence of ferroptosis induced by LBT. These results indicated that LBT is a promising USP32 inhibitor and may have favorable pharmacokinetic profiles (Figure 7). While our study identified USP32 as a critical target of LBT-induced ferroptosis via its regulation of NRF2 deubiquitination, the complexity of ferroptosis signaling suggests the existence of additional targets and pathways that may contribute to the therapeutic effects of LBT. To address this, future investigations should leverage emerging target-discovery technologies, such as PROTAC (proteolysis-targeting chimera) probe technology, which enables the selective degradation of target proteins and facilitates mechanistic exploration in dynamic cellular contexts [53]. Complementary proteomic approaches, including interactome profiling and post-translational modification analyses, could further unravel global protein networks and signaling cascades modulated by natural products [54,55]. Integrating these advanced methodologies could not only refine our understanding of natural product-driven ferroptosis but also uncover novel therapeutic vulnerabilities for ferroptosis-driven malignancies, ultimately enhancing the translational potential of natural products.

## 5. Conclusions

In summary, these results demonstrate for the first time that LBT efficiently targets USP32 and inhibits USP32-mediated NRF2 deubiquitination, thus inducing the ferroptosis of LUSC cells. Furthermore, our study provides evidence that USP32-targeted therapies may serve as efficient approaches for treating ferroptosis-driven cancers.

## Data Availability

The data that support the findings of this study are available from the corresponding author upon reasonable request.

## Supplementary Materials

The following supporting information can be downloaded at: https://www.mdpi.com/article/10.3390/cimb47030163/s1.

## Abbreviations

The following abbreviations are used in this manuscript:

USP32	ubiquitin-specific protease 32
LBT	lycobetaine
LUSC	lung squamous cell carcinoma
NSCLC	non-small-cell lung cancer
LUAD	lung adenocarcinoma
UPS	ubiquitin–proteasome system
DUBs	deubiquitinating enzymes
NRF2	nuclear factor erythroid 2-related factor 2
DARTS	drug affinity responsive target stability
MS	mass spectrometry
IP	immunoprecipitation
ROS	reactive oxygen species
GSH	glutathione
MDA	malondialdehyde
CETSA	cellular thermal shift assay
H&E	hematoxylin–eosin
Fer-1	ferrostain-1
