# Supplementary material for: Lycobetaine Has Therapeutic Efficacy in Lung Squamous Cell Carcinoma by Targeting USP32 to Trigger Ferroptosis
Source: Curr Issues Mol Biol. 2025 Feb 27;47(3):163. doi: 10.3390/cimb47030163 (PMC11941616; doi:10.3390/cimb47030163)
Supplement: Supplementary file 1 [file cimb-47-00163-s001.zip › cimb-3481833-supplementary.pdf]

Supplementary Materials for the ariticle

Lycobetaine has Therapeutic Efficacy in Lung Squamous Cell Carcinoma by Targeting USP32 to Trigger Ferroptosis

Shangping Xing [1,2,3], Hua Chai [1], Zhenlong Chen [1], Shuye Deng [4,*] and Feifei Nong [4,*]

Supplemental Materials and Methods

Quantitative real-time polymerase chain reaction (qRT-PCR)

Cellular RNA was isolated from using an RNeasy Plus Kit (Takara, Shiga, Japan). cDNA was synthesized using the Reverse Transcription System (Takara). For quantitative PCR, TB Green® Fast qPCR Mix (TaKaRa) was used. The $2^{-\Delta\Delta CT}$ method was used to analyze qRT-PCR data. The primers for RT-PCR were synthesized by the Sangon Company (Sangon, Shanghai, China). The primers sequences are as follows: USP32-F: TATACAACAGTGAGAACTACC; USP32-R: CCTTTTCTGTGGGAACCT TGTG; GAPDH-F: AGCCACATCGCTCAGACAC; GAPDH-R:GCCCAATACGAC CAAATCC.

Evaluation of the side effects of LBT in Mice

To detect the hepatorenal toxicity of LBT, the mice serums were collected to measure alanine aminotransferase (ALT), aspartate aminotransferase (AST), and creatinine (CRE) levels using ALT, AST, and CRE kits (Nanjing Jiancheng Bioengineering Institute, Nanjing, China), according to the manufacturer'sinstructions.

Supplemental Figure

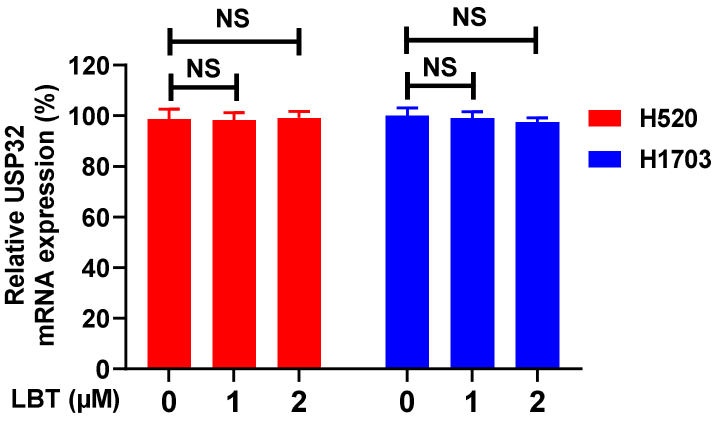

Figure S1. USP32 mRNA expression in H520 and H1703 cells treated with LBT were examined by qRT-PCR analysis. Results are presented as means ± SD. NS., no significant difference.

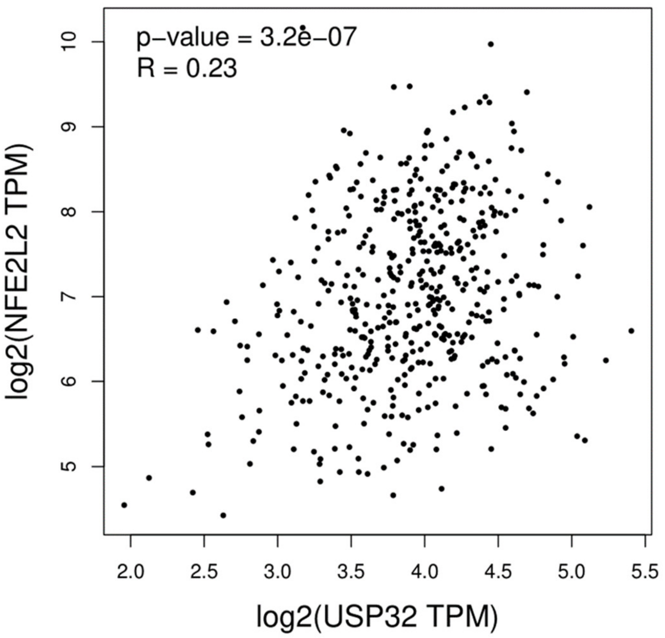

Figure S2. The correlation between USP32 and NRF2 in patients with LUSC was analyzed using the GEPIA database.

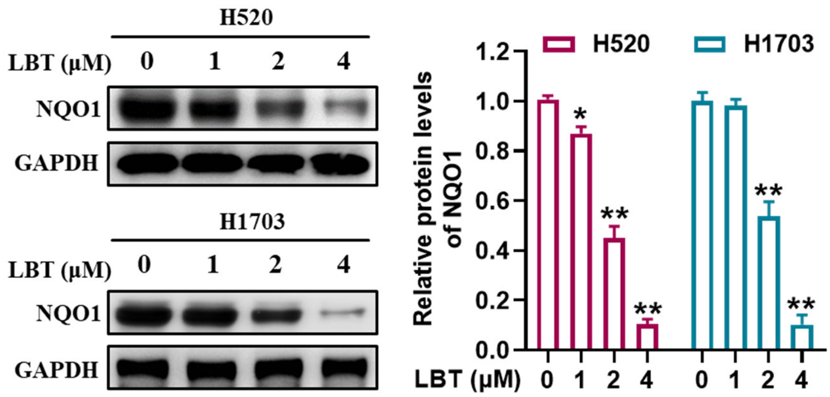

Figure S3. LBT inhibits NQO1 protein expression in LUSC cells. Protein levels of NQO1 in H520 and H1703 cells treated with LBT were examined by Western blotting. Results are presented as means ± SD. **p* < 0.05, ***p* < 0.01 versus the control group

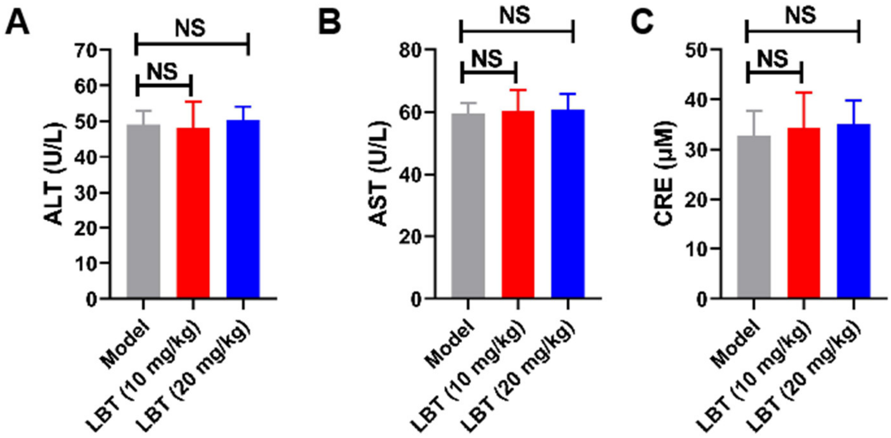

Figure S4. LBT did not exhibit toxicity in BALB/C nude mice. (A-C) The serum levels of ALT (A), AST (B), and CRE (C) in BALB/C nude mice treated with LBT were detected and calculated. Results are presented as means ± SD. NS., no significant difference.
